# Effectiveness and Suitability of Oyster Mushroom in Improving the Nutritional Value of Maize Flour Used in Complementary Foods

**DOI:** 10.1155/2021/8863776

**Published:** 2021-03-16

**Authors:** Prisca Siyame, Neema Kassim, Edna Makule

**Affiliations:** ^1^Department of Food and Nutritional Sciences, The Nelson Mandela African Institution of Science and Technology, P.O. Box 447, Arusha, Tanzania; ^2^Department of Applied Sciences, Mbeya University of Science and Technology, P.O. Box 131, Mbeya, Tanzania

## Abstract

Complementary foods based on habitual cereals such as maize have been linked with the promotion of undernutrition in young children. Blending the starchy-rich maize with nutritious-rich indigenous food such as oyster mushroom could improve the nutritional composition of complementary foods. This study investigated the effectiveness and suitability of oyster mushrooms in improving the nutritional value of maize flour commonly used as a bulk ingredient in complementary foods. Flour made of well-cleaned and sun-dried oyster mushroom was blended with maize flour at 0% (control), 30%, 40%, and 50%. Proximate composition, mineral density, and sensory evaluation were determined using standard procedures. Significant improvement in the nutritional quality of formulated flour blends with all proportions of mushroom flour was obtained . Blending maize flour with 30%, 40%, or 50% oyster mushroom flour improved the protein content of formulated flour blends from 8.63% to 18.20%, 8.63% to 20.37% and 8.63% to 22.75%, respectively. The increase in ash and fiber content ranged between 82.52% to 84.16% and 50.69% to 58.35%, respectively. Mineral content of formulated flour blends was improved from 62.89% to 64.72% (iron), 7.63% to 22.69% (zinc), 77.48% to 78.02% (calcium), and 67.55% to 67.64% (potassium). Sensory scores of porridges prepared from formulated flour blends showed good acceptance for the colour, flavour, and aroma of the porridges from three formulated flour blends. Overall, this study recommends blending oyster mushroom with maize flour to improve the nutritional content of formulated flour blend for young children who rely on maize porridge as their complementary food.

## 1. Introduction

Maize is one of the essential annual cereal crops used as a staple food after rice and wheat worldwide [1]. Maize serves as the vital basis of food security for millions of people in most developing countries, particularly in Sub-Saharan Africa [2]. In Tanzania, maize serves as the primary staple food whose cultivation covers the largest area of all cultivated land [3]. The maize consumption varies from fine maize flour blended with water to produce thin or stiff porridges, sweet composite bread, cakes, cookies, and boiling or roasting the fresh maize to make snacks and savoury dishes [4]. Furthermore, maize is the main ingredient of complementary food for young children alone or in combination with several types of cereals like sorghum, wheat, and millet [5].

White maize, predominantly used in Tanzania, is regarded as nutritionally deficient among cereals. They contain low protein levels (9.3%) than their counterparts, such as wheat and sorghum, whose protein content is above 10% [6]. Furthermore, white maize also lacks the essential amino acids, lysine and tryptophan, and is devoid of vitamin C and B group vitamins. It is also deficient in macro and trace minerals such as iron, zinc, calcium, magnesium, potassium, and phosphorus [6]. Despite its low protein and micronutrient levels, maize is the main ingredient in complementary foods for children under five years of age in Tanzania [7]. Consequently, the use of maize as the main constituent of complementary food has increased the undernutrition severity, presumably due to the grain's low nutritional profile.

To date, Tanzania is among the leading countries in Sub-Saharan Africa with a high rate of undernutrition, occurring in both forms of underweight, wasting, and stunting (TNNS, 2018). Stunting is the major form of undernutrition to young children in Tanzania, accounting for 31.8% of all cases (TNNS, 2018). The drivers of dietary-related noncommunicable diseases (NCDs), including undernutrition, are mainly unhealthy diets for both children and the elderly. Nonetheless, Tanzania is home to a wide variety of indigenous and nonindigenous crops, including pulses, nuts, cereals, fruits, and vegetables, which contain essential nutrients that contribute to healthy and productive lives. Therefore, improving the nutritional value of habitual cereal-based complementary foods such as maize using other existing crops could promote food diversification during the complementary feeding period.

Mushrooms are widely reported as ingredients of various complementary foods to promote indigenous foods as a critical contributor to nutritious complementary food. Ikujenlola and Ogunba [8] studied mushrooms as a nutritional supplement in a blend of quality protein maize and sesame—complementary food. Similarly, Mallikarjuna et al. [9] recommended using mushrooms as protein supplements to populations largely dependent on cereal diets. Aishah and Rosli [10] reported that mushroom powder is used in carbohydrate-based products to enhance nutrient composition without affecting sensory acceptance. Furthermore, Deepalakshmi and Sankaran [11] revealed that oyster mushrooms' use improved cereal-based foods' nutrient content for complementary feeding due to their enriched good nutritional profile.

Oyster mushrooms, commonly known as *Pleurotus species*, are among the most common types of cultivated mushrooms in the world [12]. This type of mushroom scores the second position among the vital mushrooms grown worldwide as they contribute approximately 2.7% of entire fresh mushrooms produced [13]. Oyster mushroom is highly nutritious as it contains a superior quality protein with entirely all essential amino acids. Besides, lysine and leucine are plentiful and always deficient in many staple cereal foods [14]. Additionally, the oyster mushrooms comprise of vital minerals such as iron, zinc, calcium, potassium, magnesium, copper, and phosphorus in a substantial amount. Mushrooms also contain abundant essential vitamins, including B group, particularly thiamine, riboflavin, nicotinic acid, folic acid, pyridoxine, pantothenic acid, and cobalamin [15]. Studies have revealed that mushrooms offer a diversity of flavour, texture, and nutrients [16].

Despite the mushrooms' potential as an excellent source of vitamins and minerals with various health and nutritional benefits, it is still underutilized in combating undernutrition, particularly stunting and micronutrient deficiency common in Tanzania. Thus, this study aimed at investigating the effectiveness and suitability of oyster mushroom (*Pleurotus ostreatus*) in improving the nutritional value of maize-based flour, habitually used as the main ingredient in complementary foods.

## 2. Materials and Methods

### 2.1. Sample Collection and Preparation

White maize grains were purchased from the central-local market in Morogoro, Tanzania. Fresh oyster mushrooms were cultivated by Okoa Oyster Mushroom Supplies and Enterprises in Morogoro, Tanzania. Collected materials (white maize grain and fresh oyster mushrooms) were immediately packed in airtight polyethylene plastic bags and transported to the Food Science Laboratory at the Sokoine University of Agriculture, Morogoro, Tanzania. Maize samples were sorted to remove extraneous materials, winnowed, dehulled, and washed thoroughly with potable water to ensure complete removal of dust and unwanted materials. The latter was followed by solar drying of maize grains to a moisture content below 10%. Similarly, fresh oyster mushrooms were sorted, washed thoroughly with potable water, sliced, and solar-dried to a moisture content below 10%. Dried maize grains and mushrooms were milled using a hammer mill and heavy-duty blender, respectively, and sieved with 1 mm sieves to obtain fine flours. Maize and mushroom flours were then blended at ratios of 100 : 0, 70 : 30, 60 : 40, and 50 : 50 *w*/*w*, respectively, to obtain maize-mushroom composite flours.

Three samples of porridges were prepared from three groups of blended maize-mushroom composite flours (70 : 30, 60 : 40, and 50 : 50 maize : mushroom flour *w*/*w*). A porridge sample from white maize flour (100 : 0 maize : mushroom flour *w*/*w*) was prepared as a control. Porridge samples were prepared by mixing 350 g of flour in 1500 ml of portable boiling water. The mixture was left to boil on a gas cooker for 15 minutes with continuous stirring. Each sample of porridge was sweetened by the addition of 30 g of brown sugar.

### 2.2. Determination of Proximate Composition

Determination of proximate composition (moisture, ash, crude protein, crude fat, and crude fiber) of maize flour, oyster mushroom flour, and the blended maize-mushroom composite flour was performed using standard official analytical methods AOAC [17].

#### 2.2.1. Crude Protein

The Kjeldahl method AOAC [17] was used to analyze crude protein content in each sample, according to Chang and Zhang [18]. Firstly, a mixture of K_2_SO_4_ (10 g), CuSO_4_ (1 g), and selenium powder (0.1 g) was prepared and used as a Kjeltec catalyst. Afterwards, 10 ml of concentrated H_2_SO_4_ and Kjeltec catalyst was added in 1 g of each sample for digestion. The mixture was heated at 420°C for 2 hours to allow the digestion of the sample. About 10 ml of 0.5 NaOH was added in 10 ml of digested samples to provide a basic condition. Following the reaction, NH_3_ was collected as NH_4_OH in a conical flask containing 20 ml of 4% boric acid and a drop of modified methyl red as an indicator. Thereafter, the distillate was titrated against 0.1 N HCL solution until the endpoint was obtained. The percentage nitrogen in the sample was determined using the following expression:

(1) $$\begin{array}{c} \%\text{Nitrogen}=\left(V_{1}-V_{2}\right)\times N\times f\times 0.014\times \frac{100}{V}\times \frac{100}{S}, \end{array}$$

where *V*_1_ is the titer for the sample (ml), *V*_2_ is the titer for blank (ml), *V* is the volume of diluted digest taken for distillation (10 ml), *N* is the normality of HCL solution, *f* is the factor for standard HCL solution, 0.014 is the milliequivalent weight of nitrogen, and *S* is the weight of the sample taken (g).

The amount of crude protein in the sample was determined by multiplying the percentage nitrogen with 6.25 as a protein conversion factor using the following expression:

(2) $$\begin{array}{c} \%\text{Protein}=\%\text{Nitrogen}\times \text{Protein factor}. \end{array}$$

#### 2.2.2. Crude Fat

The determination of crude fat in the sample was performed using standard procedure AOAC [17] with petroleum ether as an extracting solvent, as described by Nielsen and Carpenter [19]. Extraction of fat was done by placing 5 g of each sample of maize flour, oyster mushroom flour, and the blended maize-mushroom blended flours in the extraction thimble of Soxhlet apparatus (FOSS-Soxtec 2055, Denmark), followed by immersing the thimble inside the extraction containing 55 ml of petroleum ether. The extraction thimble with a fat-containing sample was heated to 60°C for 6 hours. Thereafter, solvent removal was done with a vacuum rotary evaporator's aid at 40°C, while fat drying was done in the dry oven at 70°C for 30 minutes. The amount of crude fat was obtained by subtracting an empty flask from the weight of the flask containing dried fat. The percentage of crude fat was obtained using the following expression:

(3) $$\begin{array}{c} \%\text{Fat}=\left(W_{1}-\frac{W_{2}}{W_{1}}\right)\times 100, \end{array}$$

where *W*_1_ is the weight of the sample before extraction and *W*_2_ is the weight of the sample after extraction.

#### 2.2.3. Crude Fiber

The amount of crude fiber in each sample was determined using standard procedures of AOAC [17] described by Nielsen [20]. About 2 g of each sample was added to a mixture of 200 ml of 1.25% H_2_SO_4_ and 0.31 N NaOH, boiled for 30 minutes, and washed with ethanol and petroleum ether twice. The residues obtained were then placed in clean, dry weighed crucibles and dried overnight at 100°C inside the moisture extraction oven. Thereafter, the crucibles were heated in a muffle furnace at 600°C for 6 hours, cooled, and weighed again. The weight differences of the crucibles were noted as crude fiber and calculated as percentage crude fiber as expressed as follows:

(4) $$\begin{array}{c} \%\text{Fiber}=W_{1}-\frac{W_{2}}{W_{3}}\times 100, \end{array}$$

where *W*_1_ is the weight of the sample before heating, *W*_2_ is the weight of the sample after heating, and *W*_3_ is the weight of the original sample.

#### 2.2.4. Ash Content

The ash content in each sample was determined using the AOAC [17] standard procedures described by Harris and Marshall [21]. The carbolite muffle furnace was used to heat the clean empty crucibles at 600°C for 1 h. The empty crucibles were weighed after cooling in a desiccator. The sample (2 g of each) was then placed in the crucibles, and their weight recorded, followed by burning in the muffle furnace at 550°C for 6 hours. The burnt crucibles containing samples were cooled in the desiccator and weighed again. The percentage of ash content was determined by using the following expression:

(5) $$\begin{array}{c} \%\text{Ash}=\left(W_{3}-W_{1}\right)\times \frac{100}{W_{2}}, \end{array}$$

where *W*_1_ is the weight of empty crucible, *W*_2_ is the weight of the sample, and *W*_3_ is the weight of the heated sample and the crucible.

#### 2.2.5. Moisture Content

Determination of moisture content in each sample was performed using the AOAC [17] standard procedures described by Bradley [22]. About 2 g of each sample was weighed into a dried moisture dish and placed in the moisture extraction oven (Wagtech, Germany) at 105°C for 5 hours. Thereafter, the dried samples were cooled in a desiccator and weighed again. This process was repeated thoroughly until the constant weight was attained. The percentage of moisture content of samples was calculated using the following expression:

(6) $$\begin{array}{c} \%\text{MC}=\left(\frac{W_{1}-W_{2}}{W_{1}}\right)\times 100, \end{array}$$

where *W*_1_ is the weight of the fresh sample (g) and *W*_2_ is the weight of the dry sample (g).

The percentage of moisture content (% MC) was used to obtain the dry matter content of the sample using the following equation:

(7) $$\begin{array}{c} \%\text{Dry matter}=100-\%\text{MC}. \end{array}$$

#### 2.2.6. Carbohydrate Content

The content of carbohydrate in each sample was determined by the method described by Nielsen [20]. The percentage of carbohydrate was obtained by subtracting 100 from the summation of crude protein, crude fat, crude fiber, ash, and moisture content.

(8) $$\begin{array}{c} \%\text{Total carbohydrate}=100-\%\left(\text{Protein}+\text{Fat}+\text{Fiber}+\text{Ash}+\text{Moisture content}\right). \end{array}$$

#### 2.2.7. Energy Value

The energy value was determined using the method described by Farzana and Mohajan [23]. For each blended flour sample, the energy value was obtained as the summation of crude protein, crude fat, and carbohydrate and their respective physiological values (Atwater's conversion factors) of 4, 9, and 4 calories.

#### 2.2.8. Mineral Content

The mineral (iron, zinc, calcium, and potassium) contents [24] of control maize flour and complementary flour blends were determined using atomic absorption spectrophotometer (AAS) (Thermo Scientific® iCE 3500, Waltham, USA). The AOAC [17] standard procedures were followed as described by Yeung et al. [25]. The wavelengths used in reading the absorbance of cations in the AAS were 248.3 nm for iron (Fe), 213.9 nm for zinc (Zn), and 422.7 nm for calcium. Potassium was determined using an air-liquefied petroleum gas flame on a flame photometer. The calculation of mineral content (mg/100 g) in the sample was done, as shown as follows:

(9) $$\begin{array}{c} \text{Mineral content }\left(\frac{\text{mg}}{100\text{ g}}\right)=R\times 100\text{ ml}\times \text{D}.\text{F}\left(\frac{R\times 100\text{ ml}\times \text{D}.\text{F}}{S\times 1000}\right)\times 100, \end{array}$$

where *R* is the absorbance reading in ppm, 100 is the volume of sample made, D.F is the dilution factor, 1000 is the conversion factor to mg/100 g, and *S* is the sample weight.

### 2.3. Sensory Evaluation

A total of 20 semitrained panel members comprised of nursing mothers and caregivers from Nambala Catholic Mission Dispensary in Arusha, Tanzania, were instructed to use the five-point hedonic scale for sensory evaluation of porridge. The coded maize flour porridge's sensory attributes as control and porridge from three complementary flour blend samples were evaluated using a five-point hedonic scale. The scale was in the range of 1: “dislike very much” and 5: “like very much” for the characteristics of colour, flavour, aroma texture, and general acceptability.

### 2.4. Statistical Analysis

Experiments were conducted using a completely randomized design, and statistical analysis of data performed using the Statistical Package for the Social Sciences (SPSS version 20). Data were further subjected to one-way analysis of variance (ANOVA); the main treatment means calculated from the triplicate sample analyses (*n* ≥ 3) were compared using the least significant difference (LSD) test (*p* < 0.05). All results are presented as the means ± standard deviation (SD) of multiple independent determinations.

## 3. Results and Discussion

### 3.1. Proximate Composition of Maize, Oyster Mushroom, and Complementary Flour Blends

The proximate analysis of maize flour, oyster mushroom flour, and formulated complementary flour blends is shown in Table 1. Mushroom flour had a significantly higher protein content, ash, and crude fiber (*p* ≤ 0.05), while the maize flour had a significantly higher content of crude fat, carbohydrate, and higher energy value. Similarly, formulated complementary flour blends had a significantly higher crude protein content, total ash, and crude fiber contents than those of control maize flour (*p* ≤ 0.05).

An increase in protein content of formulated complementary flour blends highlights that blending maize flours with mushrooms increased the protein content. For instance, the blending of maize flour with 30%, 40%, and 50% mushroom flour increased protein content in formulated complementary flour blends by 18.20%, 20.37%, and 22.75%, respectively. The latter is expected since the mushroom flour had high amounts of protein compared to the maize flour. These results are in proximity to those reported by Ekunseitan et al. [26], who observed an increase in protein content of cassava and wheat flour proportionately blended with mushroom flour. In a similar study, Bamidele and Fasogbon [27] reported an 83% increase in the protein content of formulated complementary flour blends of 15% oyster mushroom and 85% maize flour.

Furthermore, formulated complementary flour blends have the potential to meet the recommended daily intake (RDI) of protein from complementary foods which is 5.2 g/day at 6–8 months, 6.7 g/day at 9–11 months, and 9.1 g/day at 12–23 months of infants who are adequately breastfed [24]. This RDI can be met when a 6–8-month child will consume 75 g/meal, a 9–11-month child will consume 120 g/meal, and a 12–23-month child will consume 200 g/meal of the formulated maize-oyster mushroom complementary food. These proportions compare well with average amounts usually consumed by children of different age groups. However, the proposed feeding proportions could also be influenced by the child's appetite, the caregiver's feeding behaviour, and other characteristics such as energy density and the porridges' sweetness (Dewey and Brown, 2003). Thus, the present finding highlights that formulated complementary blends of oyster mushroom and maize as the bulk ingredient flour offer the potential for improving the nutritional level of protein for breastfed infants. Table 1 presents the proximate composition of maize, oyster mushroom, and complementary flour blends.

Ash content of formulated flour blends increased with an increase in mushroom flour in formulated flour blends. Ash content increased significantly to 7.84%, 8.11%, and 8.65% in 70 : 30, 60 : 40, and 50 : 50 maize to mushroom flour blends. These results corroborate with Bamidele and Fasogbon [27], who reported that the ash content of flour blends increased by 45.5%, 100%, and 236% as the mushroom percentage flour in maize flour increased since oyster mushroom has a higher value of ash. Similarly, Ishara et al. [28] reported 8.56% of ash for oyster mushroom flour that subsequently increased the ash content of formulated flour blends of maize and mushroom. Accordingly, the actual ash content observed in oyster mushroom flour indicates the level of minerals present [29]. Thus, the present finding showed that oyster mushrooms could improve the diet's essential minerals that promote children's health and development.

Crude fiber content increased to 15.13%, 16.73%, and 17.91% in 70 : 30, 60 : 40, and 50 : 50 maize to mushroom flour blends. The increase in ash and crude fiber contents observed in formulated flour blends could be attributed to the mushrooms' flour high fiber content. The fiber content results agree with Bamidele and Fasogbon [27], who reported a 36.4%, 90.9%, and 227.2% increase in the flour blends as the amount of mushroom flour added to maize flour increased. These findings suggest that formulated flour blends of maize and mushroom may be a good source of dietary fiber due to the renowned high dietary fiber content in mushrooms.

Crude fat content ranged from 3.81% in 70 : 30 to 4.12% in 50 : 50 formulated complementary flour blends. These results were not significantly different (*p* ≥ 0.05) from maize flour's fat content (4.95%). The results have shown that mushrooms and maize flours could be blended quite well in the ratios of 30%, 40%, or 50% without altering the fat content present in maize flour. Mushrooms are comprised of low levels of unsaturated fats such as oleic and linoleic acids but very valuable to consumer healthy [30]. Nonetheless, it is recommended that the fat content be limited to 3% to ensure its good quality. High-fat content triggers rancidity during storage, producing lousy flavour in the final flour cooked products (Ntuli et al. [31]).

The carbohydrate content in formulated complementary flour blends decreased slightly, but significantly with the increase in oyster mushroom flour ratio in the blended flour. Carbohydrate content ranged from 37.39% to 45.46% in 70 : 30 and 50 : 50 blended maize-oyster mushroom flours, respectively (Table 1). This could be attributed to low carbohydrate content (22.51%) in the mushroom flour compared to that of maize flour (68.30%). The carbohydrate content observed in oyster mushrooms was slightly lower than those found in 15 selected mushroom varieties from India, whose content ranged from 32.43% to 52.07% (Kumar et al. [32]). Similarly, the energy value of blended maize-mushroom flours ranged from 275.33 to 289.86 kcal in 70 : 30 and 50 : 50 blended maize-oyster mushroom flours, respectively. This finding highlights that maize and mushroom blending of the present study ratios results in a good balance of blended flour energy value. The latter falls within the recommended daily intake (RDI) of energy for infants aged 8 to 9 months of 94.97 kcal/kg [8]. Thus, the present formulated blended maize-oyster mushroom flours of all ratios could meet the recommended daily intake (RDI) requirement for the complementary food.

Moisture is a critical parameter to consider when accounting for the quality of flour and the acceptability of flour products. The latter is the determinant factor for shelf life and microbial growth during the storage of flour and flour products [33]. In the present study, maize flour's moisture content is 9.29%; whereas, that of formulated complementary flour blends ranged from 9.25% to 9.49% for 70 : 30 and 50 : 50 blends, respectively. These results are within the range of moisture content recommended by the World Food Programme (WFP, 2012) for maize meal flour that should not exceed 15%. Similarly, Ikujenlola and Ogunba [8] reported that solar-dried mushroom flour's moisture content ranged between 9% and 13%. The low moisture content of maize-oyster mushroom flour blends intended for complementary feeding is critical in prolonging the shelf life and maintaining microbial safety.

The present study did not look at the safety of the formulated maize-oyster mushroom blended flour, which is highly susceptible to change during storage. Literature suggests that both maize and mushroom flours are generally vulnerable to rapid moisture uptake and discoloration. Consequently, this leads to mould growth when exposed to humid conditions, thus predisposing the product to loss of quality and safety to consumers (Ojo et al., 2017). Nonetheless, documented literature also suggests that storage of maize, oyster, or blended flours in a paper bag, plastic bucket, or polyethylene bags under ambient and hot tropical weather conditions offers the chances of better shelf life stability of flour. Bamidele and Fasogbon [27] reported that the maize and oyster mushrooms flour blends' storage stability showed that the blend samples were relatively stable at room temperature (25°C) for three months with little oxidative deterioration compared to the maize flour alone. Furthermore, Han et al. (2016) revealed that oyster mushroom flour had the best storage stability properties (microbial stability, safety, nutritional, and functional properties) both at cold (4°C) and ambient (25°C) storage temperatures. Hence, this highlights that the optimum storage conditions and appropriate packaging of blended maize-oyster mushroom flour should be emphasized to end-users for maintaining the physicochemical properties and overall storage stability properties during storage.

### 3.2. Mineral Composition of Maize, Oyster Mushroom, and Complementary Flour Blends

The mineral composition of white maize, oyster mushroom, and formulated complementary flour blends is presented in Table 2. There was a significant improvement in iron, zinc, calcium, and potassium contents in blended flour attributed to mushroom flour enrichment. The iron content of blended flour increased slightly from 8.30 to 8.73 mg/100 g for 70 : 30 and 50 : 50 formulated flour blends, respectively. These results suggest that blending oyster mushroom flour with maize flour may be traditionally used as a complementary food, improving the flour blends' iron content. Ihesinachi and Eresiya [34] reported that iron is an essential micronutrient that plays a crucial role in haemoglobin formation and oxygen and electron transport in the human body and thus an essential micronutrient for complementary feeding. According to Friel et al. [35], the recommended daily intake (RDI) of iron for infants aged 12 months is 4.6 mg/day, which agrees with the iron content of formulated complementary flour blends reported in the present study.

There was a significant difference (*p* ≤ 0.05) in zinc content between the maize (3.51 mg/100 g) and oyster mushroom flours (8.31 mg/100 g). For blended flours, the zinc content increased to 3.80, 4.12, and 4.54 mg/100 g after blending maize flour with 30%, 40%, or 50% of mushroom flour, respectively. The high content of zinc observed in oyster mushrooms could have increased the zinc content in blended maize-oyster mushroom flours. Friel et al. [35] recommended that the recommended daily intake of zinc is 4 mg/day for a 12-month aged child. Thus, the present results showed that the formulated complementary flour blends could significantly contribute zinc to infants. Zinc is essential in promoting satisfactory growth and overall maintenance of the human body. Low zinc status in young children has been associated with retarded growth, poor appetite, and an impaired sense of taste [36].

The contents of both calcium and potassium observed in oyster mushroom flour were significantly higher than those observed in maize flour (*p* ≤ 0.05). The former and the latter were 4 and 6 times higher in oyster mushrooms than those observed in maize flour. Further results revealed that maize's blending with mushroom flour led to increased calcium and potassium contents in the blended flours (Table 2). The content of calcium and potassium in formulated complementary flour blends ranged between 44.59 mg/100 g to 51.13 mg/100 g and 1198.51 mg/100 g to 1214.13 mg/100 g for 70 : 30 and 50 : 50 blends, respectively. The high content of calcium and potassium in blended flours may be due to the addition of oyster mushroom flour. As previously reported by Manjunathan et al. [37], potassium composition in mushrooms could be as high as 90.8% of the total mineral content, depending on the variety. The present findings suggest that solar-dried oyster mushrooms could be a useful supplement for improving potassium and calcium contents in maize and other cereal flours.

Reports suggest that diets predominantly based on grains and legumes are of particular concern on the quantity of bioavailable micronutrients provided (Ondiek et al., 2019). Certain antinutrients such as phytate, hydrocyanides, and tannin are present in mushrooms. Nonetheless, the reported levels of these antinutrients are low and unlikely to cause any significant effect on health or to the bioavailability of the nutrients in mushrooms (Ijioma et al., 2015; Okon et al., 2015). Additionally, Ijioma et al. (2015) reported that the consumer might not experience the toxic effect of antinutrients that might be present in complementary maize-oyster mushroom blended flours due to further destruction of these substances during cooking. Furthermore, studies have revealed that maize and oyster mushroom blending does not affect the bioavailability of micronutrients present in the blended flour. For instance, Regula et al. (2010) assessed iron bioavailability from cereal products enriched with dried mushrooms. They revealed that the addition of the mushroom significantly increased the bioavailability of iron in the products. This finding highlights that the blended maize-oyster mushroom complementary food increases the iron bioavailability to malnourished children, as suggested in the present study.

### 3.3. Sensory Evaluation

The sensory evaluation results of the porridges prepared from maize flour and formulated complementary flour blends are presented in Table 3. A significant difference (*p* ≤ 0.05) in colour, flavour, aroma, texture, and overall acceptability between the control maize meal porridge and formulated complementary flour blends porridge was observed. Preference for colour, flavour, aroma, texture, and overall acceptability of samples was the highest in porridges prepared from the control maize meal flour. Formulated complementary foods developed from the three blended flours were relatively the least preferred. The porridges that were developed from 60 : 40 and 50 : 50 flour blends had relatively low ratings in most of the quality attributes, mainly colour, flavour, and aroma. Nonetheless, the panellists' assessment revealed both formulated complementary flour blends as significant.

Overall, the complementary foods developed from flour blends of maize and oyster mushrooms were not comparable to control (traditional maize flour) porridges. This could be attributed to the effect of oyster mushrooms on the flavour, aroma, and colour of the porridges developed from complementary flour blends. The formulated complementary flour blends were nutritionally improved through the enrichment with oyster mushroom, albeit there were some slight alterations in sensory attributes. Some reports revealed that enrichment of cereal flours with mushroom flour tends to decrease the sensory properties. Okafor et al. [38] observed that the ranking of scores for aroma, flavour, texture, and overall acceptability of the bread decreased by increasing mushroom powder level in an 85 : 15 wheat flour to mushroom flour blend. Therefore, blending maize flour with the lowest mushroom flour (30%) may result in complementary flour blends with improved sensory quality suitable for breastfeeding children.

## 4. Conclusion

This study has demonstrated the nutritional value of oyster mushrooms, their effectiveness, and suitability for nutritional enrichment of habitual cereal-based complementary foods, especially maize flour, for breastfeeding children. The present results have revealed oyster mushroom as an ingredient of superior nutrient density compared to maize flour. The formulation of maize flour as the bulk ingredient, enriched with oyster mushroom, improved the nutritional quality of habitual maize-based complementary foods. However, the results have also shown that the blending of oyster mushroom with the maize meal could alter the complementary porridge's sensory properties (viz. colour, flavour, and aroma). Overall, this study recommends blending oyster mushroom with maize flour to improve the nutritional content of formulated flour blend for young children who rely on maize porridge as their complementary food.

## Figures and Tables

**Table 1:** Proximate composition of maize, oyster mushroom, and complementary flour blends.

Constituent (%)	Maize flour (%)	Oyster mushroom flour (%)	Maize to oyster mushroom flour ratio		
			70 : 30	60 : 40	50 : 50
Moisture	9.29^a^ ± 0.01	9.45^b^ ± 0.03	9.25^a^ ± 0.05	9.36^a^ ± 0.04	9.49^ab^ ± 0.04
Ash	1.37^a^ ± 0.12	8.81^b^ ± 0.03	7.84^c^ ± 0.05	8.11^d^ ± 0.08	8.65^b^ ± 0.08
Crude fiber	7.46^a^ ± 0.71	22.41^b^ ± 0.01	15.13^c^ ± 0.04	16.73^d^ ± 0.23	17.91^e^ ± 0.09
Crude fat	4.95^a^ ± 0.18	0.38^b^ ± 0.02	4.12^a^ ± 0.04	3.93^a^ ± 0.08	3.81^a^ ± 0.01
Crude protein	8.63^a^ ± 0.13	36.42^b^ ± 0.50	18.20^c^ ± 0.11	20.37^d^ ± 0.50	22.75^e^ ± 0.11
Carbohydrate	68.30^a^ ± 0.75	22.51^b^ ± 0.64	45.46^c^ ± 0.06	41.50^d^ ± 0.10	37.39^e^ ± 0.03
Energy (kcal)	344.68^a^ ± 1.84	246^b^ ± 0.50	289.86^c^ ± 0.41	282.13^d^ ± 1.05	275.33^e^ ± 0.48

Mean values (*n* = 3) ± SD on a dry weight basis; means in the same row with different letters as superscript are significantly different (*p* ≤ 0.05).

**Table 2:** Mineral composition of maize, oyster mushroom, and complementary flour blends.

Constituent (mg/100 g)	Maize flour (mg/100 g)	Oyster mushroom flour (mg/100 g)	Maize to oyster mushroom flour ratio		
			70 : 30	60 : 40	50 : 50
Iron	3.08^a^ ± 0.35	12.57^b^ ± 0.12	8.30^ab^ ± 0.38	8.52^ab^ ± 0.71	8.73^ab^ ± 0.60
Zinc	3.51^a^ ± 0.27	8.31^b^ ± 0.25	3.80^ab^ ± 0.03	4.12^ab^ ± 0.28	4.54^ab^ ± 0.03
Calcium	13.33^a^ ± 3.41	49.45^b^ ± 4.22	44.59^ab^ ± 3.13	45.04^ab^ ± 4.11	51.13^ab^ ± 5.09
Potassium	391.44^a^ ± 7.22	2509.67^b^ ± 10.31	1198.51^ab^ ± 9.14	1210.48^ab^ ± 13.18	1214.13^ab^ ± 11.27

*Mean* *values* (*n* = 3) ± *SD* on a dry weight basis; means in the same row with different letters as superscript are significantly different (*p* ≤ 0.05).

**Table 3:** Sensory evaluation of porridges prepared from maize and complementary flour blends.

Attributes	Maize flour (control)	Maize to oyster mushroom flour ratio		
		70 : 30	60 : 40	50 : 50
Colour	4.25^a^ ± 0.16	3.80^b^ ± 0.14	3.75^b^ ± 0.12	3.50^b^ ± 0.18
Aroma	4.10^a^ ± 0.17	3.91^b^ ± 0.14	3.84^b^ ± 0.14	3.53^b^ ± 0.18
Flavour	4.45^a^ ± 0.10	3.95^b^ ± 0.15	3.78^b^ ± 0.13	3.57^b^ ± 0.18
Texture	4.40^a^ ± 0.10	3.95^b^ ± 0.15	3.87^b^ ± 0.15	3.75^b^ ± 0.12
Overall acceptability	4.45^a^ ± 0.10	4.05^b^ ± 0.16	3.85^b^ ± 0.15	3.78^b^ ± 0.13

Means in the same row with different letters as superscript are significantly different (*p* < 0.05).

## Data Availability

The data supporting the findings of this study are included in this research article.
